# Current Status and Potential Therapeutic Strategies for Using Non-coding RNA to Treat Diabetic Cardiomyopathy

**DOI:** 10.3389/fphys.2020.612722

**Published:** 2021-01-22

**Authors:** Amit K. Rai, Brooke Lee, Ramesh Gomez, Deepu Rajendran, Mahmood Khan, Venkata Naga Srikanth Garikipati

**Affiliations:** ^1^Department of Emergency Medicine, Institute of Behavioral Medicine and Research, Dorothy M. Davis Heart Lung and Research Institute, The Ohio State University Wexner Medical Center, Columbus, OH, United States; ^2^Department of Endocrinology, Government Medical College, Thiruvananthapuram, India; ^3^Department of Cardiology, Travancore Medical College, Kollam, India; ^4^Department of Physiology and Cell Biology, The Ohio State University Wexner Medical Center, Columbus, OH, United States

**Keywords:** diabetic cardiomyopathy, long non-coding RNA, microRNA, circular RNA, diastolic dysfunction

## Abstract

Diabetic cardiomyopathy (DMCM) is the leading cause of mortality and morbidity among diabetic patients. DMCM is characterized by an increase in oxidative stress with systemic inflammation that leads to cardiac fibrosis, ultimately causing diastolic and systolic dysfunction. Even though DMCM pathophysiology is well studied, the approach to limit this condition is not met with success. This highlights the need for more knowledge of underlying mechanisms and innovative therapies. In this regard, emerging evidence suggests a potential role of non-coding RNAs (ncRNAs), including micro-RNAs (miRNAs), long non-coding RNAs (lncRNAs), and circular RNAs (circRNAs) as novel diagnostics, mechanisms, and therapeutics in the context of DMCM. However, our understanding of ncRNAs’ role in diabetic heart disease is still in its infancy. This review provides a comprehensive update on pre-clinical and clinical studies that might develop therapeutic strategies to limit/prevent DMCM.

## Impact of Diabetes and Obesity on the Body and the Globe Currently

Obesity is presently a pandemic problem affecting both developed and developing countries (Ng et al., 2014). The projected prevalence of obesity in adults in the United States is estimated to be 49.8%, indicating that one in two individuals will have obesity (Ward et al., 2019). The United States’s most common body mass index (BMI) category will be severe obesity by 2030. In children and adolescents, obesity has risen 10-fold over the past 40 years, taking 124 million from 11 million (World Health Organization, 2018). Though the rise in children’s BMI has plateaued over decades, the trend is still alarming in South Asia, the Middle East, and North Africa, where the prevalence is more than 20% (Abarca-Gómez et al., 2017). In children with class 3 obesity, less than 26 h of intensive behavioral therapy mechanisms, lasting between 6 and 12 months, was shown to be associated with higher systolic and diastolic blood pressures, higher odds for hypertension, type 2 diabetes mellitus (T2DM), and obstructive sleep apnea (Tsao-Wu et al., 2019). The overweight trend in adolescence is associated with higher cardiovascular (CV) and all-cause mortality in men after a 55-year follow-up (Must et al., 1992).

Large community-based follow-up studies have shown that a higher BMI is associated with heart failure (HF), more for women (7%) compared with men (5%) (Kenchaiah et al., 2002). The duration of obesity is important and for every two additional years living with obesity, the hazard ratio for all-cause and CV mortality was 1.06 and 1.07, respectively (Kenchaiah et al., 2002). The relative risk of CV death among the heaviest individuals was 1.57 in comparison with the category with the lowest BMI in meta-analytic studies (Jiang et al., 2013). Within different classes of obesity, independent of gender, smoking status, and educational level, CV mortality was 1.29 among class 1, 1.87 among class 2, and 2.2 among class 3 of obesity (Jiang et al., 2013). Obesity and hyperglycemia in C57BL/6J *db*/*db* mice were found to be associated with hypertrophy of the cardiac myocyte, thickening of the perimysial collagen, vascular rarefaction, and mild fibrosis of the endomysium and perivascular region, mainly by the addition of the extracellular matrix via fibroblasts in the interstitium (Alex et al., 2018).

A meta-analysis showed that an obesity paradox exists in HF, and the nadir was seen in overweight subjects (Mahajan et al., 2020). The obese and overweight subjects, classified based on their pre-HF BMI, had lower mortality rates when admitted for HF (Pokharel et al., 2017). Mahajan et al. (2020) also showed that intentional weight loss in obese subjects without established HF was associated with significant improvement in left ventricular (LV) diastolic dysfunction and reduced LV size. Though there is a decline in the incidence of T2DM in the United States, which is primarily attributed to the decline in Hispanic whites, the global prevalence of diabetes among adults over 18 years of age rose from 4.7% in 1980 to 8.5% in 2014, with a 5% increase in premature mortality due to diabetes between 2000 and 2016 (Centers for Disease Control and Prevention, 2020). South Asians have a high-fat percentage for any given BMI, making them vulnerable to metabolic diseases (Lear et al., 2007). About 19 million adults ages 20–79 were living with diabetes in the International Diabetes Federation of Africa Region in 2019, and the number is estimated to increase to 47 million by 2045. Another 45 million adults (20–79) in this region have impaired glucose tolerance (IGT), and the figure is expected to reach 110 million by 2045 (Cho et al., 2018).

**FIGURE 1:**
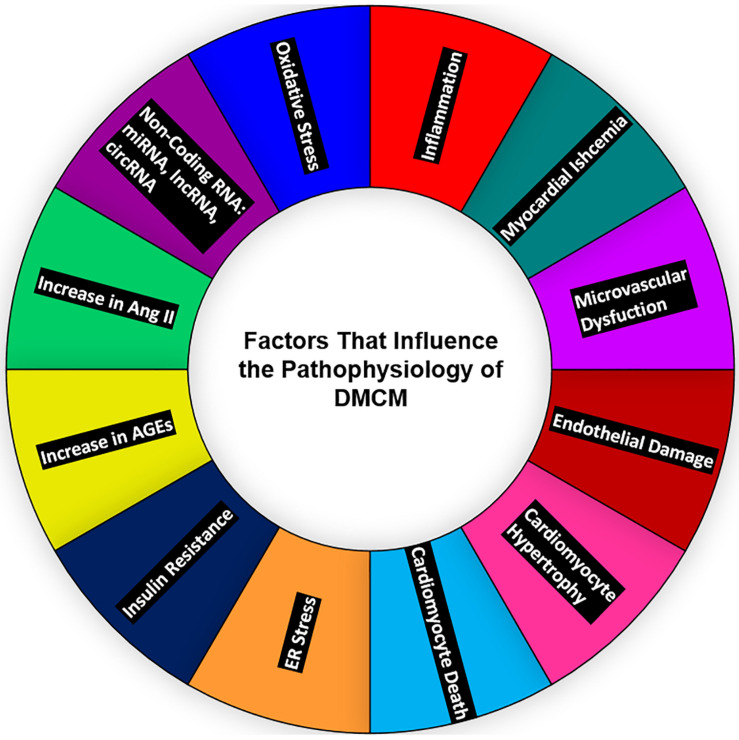
Major contributors to diabetic heart disease—oxidative stress, ncRNAs; miRNA, lncRNA, circRNA, the upregulation of Ang-II and AGEs, insulin resistance, ER stress, cardiomyocyte death, cardiomyocyte hypertrophy, endothelial damage, microvascular dysfunction, myocardial ischemia, and inflammation—all affect the heart and contribute to the development of DMCM. ncRNAs, non-coding RNAs; miRNAs, micro-RNAs; lncRNA, long non-coding RNA; circRNA, circular RNA; Ang II, angiotensin II; ER, endoplasmic reticulum; DMCM, diabetic cardiomyopathy.

Diabetic cardiomyopathy (DMCM) denotes LV dysfunction in diabetic subjects (Figure 1) with or without preserved ejection fraction (pEF) due to interstitial fibrosis and repercussions of LV hypertrophy caused by a myriad of factors (Lorenzo-Almoros et al., 2017). Leyden was the first to postulate that diabetes can cause HF in his journal, *Asthma, and Diabetes Mellitus*, in as early as 1881 (Leyden, 1881). Shirley Rubler and team first used the term DMCM and HF in the absence of significant coronary artery disease in four patients with T2DM and glomerulosclerosis. There is a two- to four-fold increase in HF incidence rates in diabetic individuals compared with non-diabetic individuals (Dunlay et al., 2019). The histopathology revealed diffuse fibrotic strands extending between myocardial muscle bundles and myofibrillar hypertrophy (Rubler et al., 1972). The strong heart study showed that subjects of T2DM had statistically significant, 6–12%, higher LV mass corrected for body surface area, inter-ventricular and posterior wall thickness, 5% lower stress corrected mid-wall shortening, and arterial stiffness as demonstrated by a 13% increase in pulse pressure to stroke volume ratio (Devereux et al., 2000). Streptozotocin (STZ)-induced diabetes in male Sprague–Dawley (SD)-derived rats, which led to lipid accumulation in the myocardium associated with a breach in myocardial integrity, characterized by the loss of contractile proteins, sarcoplasmic reticulum swelling, myocytolysis, the formation of myelin, and contractual bands (Jackson et al., 1985). There was a decrease in myosin ATPase enzyme in the myocardium, with the slow V3 electrocardiogram electrode isoform predominating as early as 2 weeks after the induction of diabetes (Malhotra et al., 1981). A 1 mmol/L increase in fasting plasma glucose was associated with a 1.23-fold increase in risk for hospitalization for HF (Held et al., 2007). Rising glycosylated hemoglobin was associated with a higher hospitalization rate for HF in middle-aged subjects without diabetes (HbA1c 5.5–6%), implicating that HF begins even before the development of overt diabetes (Matsushita et al., 2010). Doppler studies showed diastolic dysfunction in up to 33% of young asymptomatic diabetic subjects (Zarich et al., 1988). Animal models provided insights that the presence of hypertension can aggravate the myocardial injury in rat models, evidenced by a greater heart weight to body weight ratio and severe interstitial fibrosis than diabetes or hypertension alone (Factor et al., 1981). Another study conducted in veterans with diabetes showed mortality to follow a U-shaped pattern with the achieved HbA1c level (7.1% to 7.8%) (Aguilar et al., 2009). In India, diabetes prevalence varies between 5% and 10% as per the gross domestic product of states, and a large proportion of patients (47.3%) diagnosed with diabetes during the study were unaware of their diabetic status (Anjana et al., 2017). The maternal nutrition study conducted in India showed that the babies born in an unfavorable intrauterine environment are thinner and have smaller muscle mass and preserved subcutaneous fat than their European counterparts (Yajnik et al., 2003). A subsequent defect in calcium transportation in the isolated sarcoplasmic reticulum has been recorded in DMCM (Ganguly et al., 1983). While treatment with insulin showed reversibility of alterations in myocardial function, contractile protein biochemistry was as early as 10 days of therapy (Fein et al., 1984).

## The Treatment Approach to Type 1 Diabetes Mellitus and Cardiomyopathy

Type 1 diabetes mellitus (T1DM) is an autoimmune disease in which pancreatic β cells that produce insulin are destroyed. The absence of β cells/insulin impairs breakdown of sugar in the bloodstream and use it for energy (DiMeglio et al., 2018). Using STZ-induced diabetes rat models (Malhotra et al., 1981), Malhotra et al. (1981) showed the efficacy of intensive glycemic control in mitigating the structural and molecular changes in cardiomyopathy. Abnormal diastolic functioning correlated with glycemic control in T1DM subjects (Shishehbor et al., 2003). When T1DM subjects were intensively treated thrice or four times with insulin daily, the reduction of inflammatory markers was not reassuring (Schaumberg et al., 2005). There was a decrease in soluble intercellular adhesion molecule type 1 (sICAM). Still, soluble tumor necrosis factor-α receptor 1 (sTNR-R1) and high-sensitivity C-reactive protein (hs-CRP) had increased, especially in subjects who gained weight (Schaumberg et al., 2005). There was an improvement in LV echocardiographic parameters: peak lengthening rate, the peak wall thinning rate, E/A ratio, and reduction in the E wave deceleration time (Grandi et al., 2002). Another study failed to replicate these findings but has found a reduction in LV mass and interventricular thickness irrespective of diabetic nephropathy (Aepfelbacher et al., 2004; Weinrauch et al., 2005; Grandi et al., 2006). The Diabetes Control and Complication Trial (DCCT)/Epidemiology of Diabetes Interventions and Complications (EDIC) study, aimed at intensive glycemic control in T1DM subjects, showed a significant reduction in any CV disease (CVD) by 30% and major CV events by 32% in the initial 6.5 years of follow-up. The effect on HF prevention was not specifically looked upon (Control and Trial, 2016). Angiotensin-converting enzyme (ACE) inhibitors effectively reduced mortality and prevented hospitalization for HF in T1DM (Lewis and Lewis, 2004). The SGLT2 inhibitors empagliflozin and ipragliflozin normalized the endothelium of STZ-induced rat models, which may prove useful in human subjects if used along with a reasonable dosage of insulin (Oelze et al., 2014; Salim et al., 2016). Prospective randomized clinical trials are needed to assess whether this translates to clinical benefits.

## Type 2 Diabetes Mellitus and Cardiomyopathy Management: Evidence and Prospects

Type 2 diabetes mellitus is a metabolic disorder, unlike T1DM. β cells are not destroyed; they still produce insulin, but T2DM subjects are resistant and cannot respond to insulin (Fletcher et al., 2002). ACE inhibitors, angiotensin receptor blockers, aldosterone antagonists, spironolactone, and β blockers are the most common medications used to treat HF in individuals with or without diabetes. The recent addition to the armamentarium is the valsartan/sacubitril combination. Valsartan–sacubitril, compared with enalapril, significantly prevented death due to CVD and all-cause mortality, while also reducing the number of hospitalizations for HF by 21% in subjects with reduced ejection fraction (PARADIGM–HF trial) (McMurray et al., 2014). However, it failed to achieve any significant benefits in subjects with pEF compared with valsartan alone (PARAGON-HF trial) (Solomon et al., 2019). High fasting plasma glucose and HbA1c predicted the rate of hospitalizations for HF in subjects with abnormal glucose regulation. Intensive glucose lowering with insulin and sulfonylureas did not improve the CV outcomes. Despite achieving adequate glycemic control, HbA1c reduction was not shown to be associated with improvement in CV mortality in the United Kingdom Prospective Diabetes Study (UKPDS) and the ADVANCE Collaborative Group trial (Group, 1998; Advance Collaborative Group, Patel et al., 2008). The 15-year follow-up study of the Veterans Affairs Diabetes Trial (VADT) showed no legacy effect of initial intensive glycemic control. This study showed prevention of CV events during the initial and prolonged phase of the study, but not in the long-term 15 years follow-up (Reaven et al., 2019). Another large meta-analysis of eight trials with 37,229 subjects showed that intensive glycemic control was not associated with lower incidences of HF (Castagno et al., 2011). Pioglitazone use was associated with a decline in significant CV events including stroke, fatal, and non-fatal myocardial infarction (MI), but it was associated with a higher incidence of HF due to renal sodium retention in the PROACTIVE study (Dormandy et al., 2005; Kernan et al., 2016). Though there was a higher incidence of edema in the IRIS study, subjects who experienced a stroke and had insulin resistance did not show an increased risk of HF (Dormandy et al., 2005; Kernan et al., 2016). Acarbose prevented new-onset diabetes, hypertension, and CV adverse events in subjects with IGT in the Study to Prevent Non-Insulin Dependent Diabetes (STOP-NIDD) but did not reduce the incidence of HF in the ACE study (Delorme and Chiasson, 2005; Holman et al., 2017b). Metformin improved passive stiffness of the LV chamber and preserved exercise capacity in rat models experiencing HF with pEF (HFpEF) (Slater et al., 2019). A recent retrospective study conducted in Taiwan revealed that metformin usage in T2DM was associated with a 40% decrease in hospital admissions for HF (Tseng, 2019). A meta-analysis of 40 studies by Han et al. (2019), comprising more than 10,000,000 subjects, showed that metformin use for MI and HF was associated with reduced mortality. The incidence of CV events in HF was also reduced (adjusted hazard ratio: 0.79/0.83/0.84, respectively) in subjects with diabetes, but not in subjects without diabetes (Han et al., 2019). The activation of AMP kinase (AMPK) by metformin was shown to inhibit the TNF-α expression. The upregulation of the ABCG1 gene attenuates the conversion of a monocyte-macrophage into a foam cell (Hattori et al., 2006; Isoda et al., 2006); knockdown of AMPK-α abolishes this anti-inflammatory effect of metformin. AMPK activation prevents cardiac hypertrophy predominantly by inhibiting *O*-GlcNAcylation of cardiac troponin T when inhibiting the phosphorylation of the same glutamine:fructose-6-phosphate aminotransferase enzyme (GFAT) (Gélinas et al., 2018). DPP4 inhibitor saxagliptin (SAVOR-TIMI 53) increased hospitalization for HF in patients with diabetes and established CVD or multiple CV risk factors. While sitagliptin (TECOS), alogliptin (EXAMINE), and linagliptin (CARMELINA) were all found to be safe, with regard to HF (Packer, 2018). DDP4 inhibition causes sympathetic nervous system overactivity via enhanced stromal cell-derived factor-1 (SDF-1), neuropeptide Y, and substance P leading to myocyte apoptosis (Packer, 2018). Though DPP4 inhibitors have received a green signal, these studies give a cautionary notice to clinicians. At the same time, they opt for this class of medications, especially in subjects with a high risk for HF.

In small-scale studies, glucagon-like peptide 1 (GLP-1) analog infusion was associated with improved LV function in HF subjects. The LEADER trial showed a non-significant, 13% reduction in HF hospitalizations, but the FIGHT trial did not offer post-admission stability in subjects admitted with HF and reduced ejection fraction (Margulies et al., 2016; Marso et al., 2016b). Experimental studies showed liraglutide could render energy-deprived cardiomyocytes and lead to cardiac function deterioration (Shiraki et al., 2019). However, in clinical trials, GLP-1 agonists dulaglutide, albiglutide, and injectable/oral semaglutide showed a significant reduction of CV events than did the placebo (Marso et al., 2016a; Hernandez et al., 2018; Gerstein et al., 2019; Husain et al., 2019). Lixisenatide and weekly exenatide did not show any CV beneficial advantage over the placebo (Pfeffer et al., 2015; Holman et al., 2017a).

Recent evidence suggests the use of novel dual glucose-dependent insulinotropic polypeptide (GIP) and GLP-1 receptor agonist play a beneficial role in patients of T2DM (Holst and Rosenkilde, 2020). In pre-clinical trials, LY3298176, a novel dual GIP and GLP-1 receptor agonist, demonstrated activation of both receptors (GIP and GLP-1) led to insulin secretion. *In vivo* study revealed glucose tolerance and reduced body weight and food intake in GLP-1R and GIPR null C57BL/6 mice. These cumulative effects were significantly higher with the comparison GLP-1 agonist alone, suggesting that activation of both GIP and GLP-1 receptors is crucial for controlling blood glucose level and body weight. Further, Coskun et al. (2018) performed a phase 1 clinical trial using LY3298176 on T2DM patients and found a significant decrease in fasting blood glucose.

Further, the same group took the drug LY3298176 to a phase 2 clinical trial and found better management of glucose levels and significant weight loss in T2DM patients than dulaglutide (Frias et al., 2018). These results highlight a novel dual incretin receptor agonist’s promising role as a better drug candidate in managing T2DM patients. Future studies are warranted to validate this novel dual incretin receptor agonist in the management of DMCM.

The EMPA-REG outcome trial’s dramatic results were that the SGLT2 inhibitor empagliflozin showed a 35% reduction of HF in subjects with established CVD. This brought a paradigm shift in the management of glycemic control (Zinman et al., 2015). The CANVAS (canagliflozin) and the DECLARETIMI 58 (dapagliflozin) trial followed a similar decline in hospitalizations for HF in subjects with and without established CVD (Neal et al., 2017; Wiviott et al., 2019). The HF trials done subsequently in subjects with or without diabetes showed a statistically significant, 25% reduction in HF hospitalization, with reduced ejection fraction by empagliflozin (EMPEROR-REDUCED trial) (McMurray et al., 2019). The study subjects had severe LV dysfunction and were on optimal anti-failure medications, including aldosterone antagonists and neprilysin inhibitors. The DAPA-HF (dapagliflozin) trial (in which the enrolled subjects had a better median ejection fraction and were not on aldosterone antagonists nor neprilysin inhibitors) also showed a reduction in HF events irrespective of their diabetic status (Packer et al., 2020). SGLT2 inhibitors are more promising than any other medication group in managing diabetes and HF’s deadly duo. The key mechanism by which SGLT2 inhibitors modifies HF risk is that they bind the sodium binding sites of sodium-hydrogen exchanger (NHE-1) and attenuate the cytosolic sodium and calcium, improving calcium concentration in the sarcoplasmic reticulum (Uthman et al., 2018). Dapagliflozin attenuates inflammasome NRLP3, mediates inflammation, and improves hyperglycemia-induced LV dysfunction in mouse models (Ye et al., 2017).

SGLT1 is a vital transporter of glucose in the myocardium, and its overexpression was associated with pathologic cardiac hypertrophy, while its knockdown attenuated cardiomyopathy in PRKAG2 knockout murine models (Banerjee et al., 2009; Ramratnam et al., 2014). In db/db mice models, the SGLT1 inhibitor, phlorizin, preserved the typical cardiac structure (Cai et al., 2013). The first dual SGLT1 and SGLT2 blocker, sotagliflozin, improves postprandial glucose in patients with diabetes (Powell et al., 2020). Whether sotagliflozin and similar molecules will have an added advantage over selective SGLT2 inhibitors in the prevention and management of DMCM needs to be further investigated (Table 1).

**TABLE 1:** List of clinical trials associated with treatment of DMCM.

**Name of the trial (number of subjects)**	**Molecule studied (dose and route of administration)**	**MACE HR (CI/p value)**	**CV death**	**Heart failure HR (95% CI)**	**Outcomes**	**Limitations of study**	**References**
PARADIGM-HF (8442)	LCZ696 (200 mg twice daily) and enalapril (10 mg daily)	0.80 (0.73–0.87) *p* < 0.001	0.80 (0.71–0.89) *p* < 0.001	0.79 (0.71–0.89) *p* < 0.001	LCZ696 performed better in comparison of enalapril to reduce risk of death and hospitalization in heart failure	LCZ696 showed the hypotension in patients	McMurray et al. (2014)
PARAGON-HF (4822)	Sacubitril–valsartan (97 mg sacubitril and 103 mg valsartan twice daily) and valsartan (160 mg twice daily)	0.87 (0.75–1.01)	0.95 (0.79–1.16)	0.85 (0.72–1.00)	Sacubitril–valsartan did not significantly lowered the risk of death due to HF and hospitalization in heart failure	Did not shown any cardiovascular benefits in patients of heart failure with preserved ejection fraction	Solomon et al. (2019)
PROACTIVE STUDY (5238)	Pioglitazone (15–45 mg orally OD)	0.96 (0.78–1.18)	NA	NA	Reduced the all kind of mortality including non-fatal MI and stroke in T2DM patients	NA	Dormandy et al. (2005)
LEADER (9340)	Liraglutide (1.8 mg s/c OD)	0.87 (0.78–0.97) *p* < 0.015	0.78 (0.66–0.93) *p* < 0.007	0.87 (0.73–1.05) *p* < 0.14	Liraglutide reduces the event of death due to cardiovascular causes and non-fatal stroke T2DM patients with the comparison of placebo group	Safety and efficacy data need further validation due to short time period of study on patients (3.5–5 years study)	Marso et al. (2016b)
SUSTAIN-6 (3297)	Semaglutide (0⋅5 or 1 mg s/c per week)	0.74 (0.58–0.95) *p* < 0.016	0.98 (0.65–1.48) *p* < 0.92	1.11 (0.77–1.61) *p* < 0.57	Semaglutide significantly reduces the event of cardiovascular death and non-fatal MI in patients of T2DM with the placebo group	Patients were studied for shorter time period (2.1 years) and events of gastrointestinal abnormality reported	Marso et al. (2016a)
EXSCEL (14752)	Exenatide (2 mg s/c weekly)	0.91 (0.83–1.00) *p* < 0.061	0.88 (0.76–1.02) *p* < 0.096	0.94 (0.78-1.13)	No significant difference in key adverse cardiovascular occurrence in both groups (exenatide vs. placebo)	Loss to follow up rate was high. First generation injection device was complex. There was no run-in period determined by researcher. Not a standardized method of care	Holman et al. (2017a)
Harmony outcomes (9463)	Albiglutide (30 or 50 mg s/c weekly)	0.78 (0.68–0.90) *p* < 0.0001	0.93 (0.73–1.19) *p* < 0.58	0.85 (0.70–1.04) *p* < 0.113	Reduced the potential of CV complications. Improved cardiovascular outcomes for T2DM patients.	Short follow up. 25% of participants discontinued the study prior to completion. Microvascular complications were not noted. Urinary albumin excretion and lipids were no measured.	Hernandez et al. (2018)
REWIND (9901)	Dulaglutide (1.5 mg per week s/c)	0.88 (0.79–0.99) *p* < 0.026	0.91 (0.78–1.06) *p* < 0.21	0.93 (0.77–1.12) *p* < 0.46	Lowers CV outcomes within 5 years. Lowers blood pressure. Increases weight loss. Lowers glucose levels while preventing risk of hypoglycemia	Over 25% of participants stopped using dulaglutide before study was completed.	Gerstein et al. (2019)
EMPA-REG (7020)	Empagliflozin (10–25 mg once daily)	0.86 (0.74–0.99)	0.62 (0.49–0.77) *p* < 0.001	0.65 (0.5–0.85) *p* < 0.002	Lowered the rate of primary composite CV outcome and death from any cause when studying the drug added to standard care	The discontinuation rate of both groups very similar	Zinman et al. (2015)
DAPA-HF (4744)	Dapagliflozin (10 mg once daily)	0.74 (0.65–0.85) *p* < 0.001	0.82 (0.69–0.98)	0.70 (0.59–0.83)	Reduces the risk of worsening CV conditions compared to the placebo. The adverse event rate did not differ	Inclusion and exclusion criteria were very particular which could have decreased the generalizability of the study. The study also had limited diversity, also lowering generalizability	McMurray et al. (2019)
CANVAS PROGRAM (10142)	Canagliflozin (100 mg and 300 mg orally daily)	0.86 (0.75–0.97) *p* < 0.60	0.9 (0.7–1.15)	0.67 (0.52–0.87)	Decreases risk of mortality from CV complication, non-fatal MI and non-fatal stroke. Increased risk of amputation	Low levels of end-stage renal disease. Increase use of glucose-lowering solutions in the placebo group may have resulted in risks or benefits of Canagliflozin	Neal et al. (2017)
EMPEROR-reduced (3730)	Empagliflozin (10 mg daily)	0.75 (0.65–0.86) *p* < 0.001	0.92 (0.75–1.12)	0.69 (0.59–0.81)	Reduction in the risk of CV death or hospitalization for HF compared to the placebo. Slower progression of renal failure with chronic HF and reduced ejection fraction.	Uncomplicated genital tract infection was found higher in empagliflozin treated group	Packer et al. (2020)
DECLARE-TIMI 58 (17,160)	Dapagliflozin (10 mg daily)	0.93 (0.84–1.03) *p* < 0.17	0.98 (0.82–1.17)	0.73 (0.61–0.88)	Reduction in the hospitalization of the patients with heart failure	Genital tract infection were higher in treated groups that leads to discontinuation of study	Wiviott et al. (2019)

*MACE, major adverse cardiovascular events; HR, hazard ratio; CI, confidence interval; CV, cardiovascular; mg, milligram; s/c, sub-cutaneous; OD, once daily; T2DM, type 2 diabetes mellitus; MI, myocardial infarction; HF, heart failure; NA, not applicable.*

Overall, future studies should identify a potential pharmacological agent that improves insulin resistance and β cell dysfunction, does not induce hypoglycemia, attenuates inflammation, and improves the myocardium’s fuel kinetics. This strategy seems to be the brightest potential in preventing DMCM. Further, that pharmacological agent should not activate renin–angiotensin or the sympathetic nervous system. Thus, a comprehensive treatment plan is of utmost importance.

### Role of Non-coding RNAs in Diabetic Cardiomyopathy

Diabetic cardiomyopathy can be characterized by many events such as mitochondrial dysfunction, inflammation, oxidative stress, apoptosis, autophagy, microangiopathy, and myocardial metabolic abnormalities (Tan et al., 2020). However, no existent approaches efficiently limit/reduce the development of DMCM (Table 2) (Swedberg et al., 2010; Insulin, 2012; Eurich et al., 2013; Green et al., 2015; Zinman et al., 2015; Ponikowski et al., 2016; Jorsal et al., 2017; Garber et al., 2018). Despite development in controlling glycemic levels, CV events in diabetic patients significantly burden patients’ health-care costs, quality of life, and mortality (Borghetti et al., 2018). This emphasizes a need for novel diagnostics, more understanding of underlying mechanisms, and new therapeutic strategies.

**TABLE 2:** Current therapies of DMCM.

**Category of drug**	**Name**	**Mode of action**	**References**
Biguanides	Metformin	Controls the blood glucose levels by activating the AMPK pathway	Eurich et al. (2013) and Garber et al. (2018)
Insulin	Humulin	Increases glucose uptake in insulin sensitive tissues and lowers peripheral blood glucose	Insulin (2012)
Angiotensin-converting enzyme inhibitor	Ramipril	angiotensin convertase enzyme inhibitor (ACE-i)	Ponikowski et al. (2016)
Angiotensin receptor neprilysin inhibitor	Sacubitril/Valsartan	angiotensin II receptor blocker/neprilysin inhibitor	Ponikowski et al. (2016)
Ivabradine		Decreases the heart rate by suppressing the I_*f*_ channel in the sinus node	Swedberg et al. (2010)
DPP4 inhibitor	Sitagliptin	Blocks the enzyme DPP4 and stimulates insulin secretion	Green et al. (2015)
GLP1 receptor agonist	Exenatide, Liraglutide	Release the insulin from pancreatic-beta cell	Jorsal et al. (2017)
SGLT2 inhibitor	Empagliflozin	Reduces blood glucose level by acting on kidney to remove sugar from body to urine.	Zinman et al. (2015)

**FIGURE 2:**
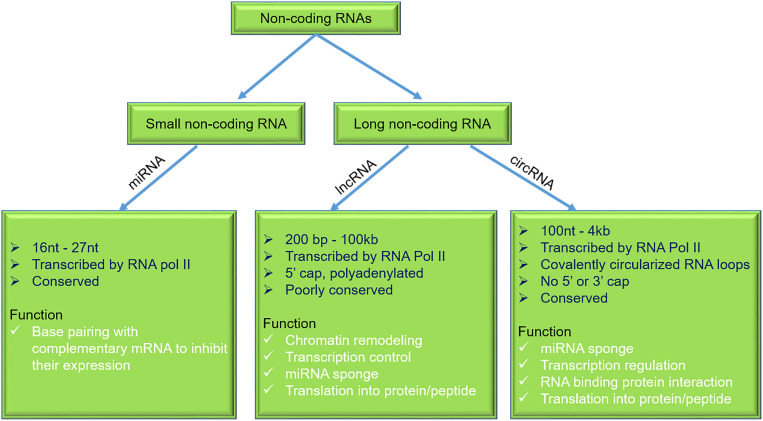
General characteristics and functions of miRNAs, lncRNAs, and circRNAs. miRNAs, micro-RNAs; lncRNAs, long non-coding RNAs; circRNAs, circular RNAs.

Emerging evidence suggests that non-coding regions of the human genome have a significant role in the health and disease pathophysiology (Esteller, 2011). So far, in the human genome, well-studied sequences are protein-coding genes, which account for only 1.5–2% of the total genome, while the majority of the genome is shared by the non-coding RNAs (ncRNAs). The majority of ncRNAs can be categorized into small and long ncRNA (lncRNA) (Zhang et al., 2019). This review focuses on the role of micro-RNAs (miRNAs), lncRNAs, and circular RNAs (circRNAs) in the DMCM (Figure 2 and Table 3).

**TABLE 3:** Role of ncRNAs in diabetic cardiomyopathy.

**Source cell/animals**	**nc-RNA**	**Target delivery method**	**Targets**	**Outcome**	**References**
Cardiac myocytes	miR-133	Lentivirus induced transduction (*in vitro*)	Inhibits GLUT4 via KLF15	Reduces insulin stimulated glucose uptake in cardiomyocyte	Horie et al. (2009)
Cardiac microvascular endothelial cells/diabetic mice	miR-200b	miR-200b mimic or antagomir (*in vitro*)	VEGF, zinc finger E-box–binding homeobox, TGF-β1, and p300	Prevents diabetes induced changes in structure and function of heart	Feng et al. (2016)
Primary cardiac fibroblasts	miR-21	miR-21 mimic/miR-21 inhibitor (*in vitro*)	Dual specific phosphatase8	Promotes HG induced cardiac fibrosis	Liu et al. (2014)
HG treated cardiomyocyte	miR-133a	miR-133a mimic (*in vitro*)	SGK1, IGF1R and MEF2	Prevents HG induced cardiomyocyte hypertrophy	Feng et al. (2010)
STZ-induced diabetic mice (miR-133a Tg mice)	miR-133a	miR-133a Tg mice (over expression in heart)	EP300, TGF-β, FN1 and COL4A1	Prevents diabetes induced cardiac fibrosis	Chen et al. (2014)
STZ induced SD rats/miR-133a Tg mice	miR-133 a	Lentivirus mediated miR-133a/anti-miR-133a (*in vivo*)	Tyrosine aminotransferase and tyrosine hydroxylase	Increases contractility of heart	Nandi et al. (2016)
HG treated neonatal rat cardiomyocyte	miR-200c	miR-200c inhibitor (*in vitro)*	DUSP1, JNK1, ERK and p38	Increases cardiac hypertrophy	Singh et al. (2017)
HG treated cardiomyocyte and STZ induced diabetic mice	miR-144	miR-144 mimic/miR-144 inhibitor (*in vitro* and *in vivo*)	Rac1, AMPK phosphorylation and PGC-1α	Reduces cardiac apoptosis	Tao et al. (2020)
HG treated cardiomyocyte/STZ treated SD rat	miR-223	miR-223 inhibitor (*in vitro* and *in vivo)*	NLRP3 inflammasome	Induces cardiac fibrosis and apoptosis	Xu et al. (2020)
HG treated H9c2 cells/STZ treated mice fed HFD	mi-RNA 22	mi-RNA 22 overexpression by intravenous injection of adenovirus (*in vivo*)	Sirt1	Attenuates oxidative stress	Tang et al. (2018)
HFD fed mice	miR-19b-3p, miRNA181b-5p	NA	NA	Cardiac hypertrophy	Copier et al. (2017)
MMEC of T2DM Goto-Kakizaki rats	miR-193-5p	miR-193-5p antagomir (*in vitro*)	IGF2	Promotes angiogenesis	Yi et al. (2017)
Neonatal cardiac myocyte treated with palmitic acid/HFD fed mice	miR-451	Cardiomyocyte-specific miR-451 knockout mice	LKB1/AMP activated protein kinase pathway	Promotes lipotoxicity in cardiomyocyte and increases cardiac hypertrophy	Kuwabara et al. (2015)
db/db mice and diabetic patients	miR-320	miR-320 mimic/miR-320 inhibitor (*in vitro* and *in vivo*)	CD36	Induces lipotoxicity in diabetic heart	Li et al. (2019)
db/db mice	miR-126, miR-499, miR-15b	miR-126 mimic and anti-miR-126 (*in vivo*)	VEGF and SPRED-1	Increased coronary arterioles and improved cardiac dysfunction	Lew et al. (2020)
STZ induced diabetes in rats	lncRNA-MALAT1	Intracoronary injection with lentivirus MALAT1 shRNA/scramble shRNA (*in vivo*)	NA	Induces cardiomyocyte apoptosis/left ventricular function	Zhang et al. (2016)
Cultured neonatal rat cardiomyocyte/STZ treated rats	lncRNA- H19	Intracoronary injection with lentivirus pcDNA-H19 (*in vivo*)	VDAC1	Suppresses cardiomyocyte apoptosis	Li X. et al. (2016)
Mouse neonatal cardiac fibroblast/HFD fed mice and also STZ induced diabetes	lncRNA-Crnde	Injected with AAV-Crnde or AAV-shCrnde (*in vivo*)	Smad3	Attenuates cardiac fibrosis	Zheng et al. (2019)
AC16/cardiomyocyte cells, STZ induced diabetes	lncRNA-Kcnq1ot1	si-Kcnq1ot1 and Kcnq1ot1 lentivirus-shRNA (*in vitro* and *in vivo*)	miR-214-3p and caspase-1	Promotes pyroptosis in DMCM	Yang et al. (2018)
T2DM patients	lncRNA-LIPCAR, SENCAR and MIAT	NA	NA	Left ventricular diastolic function	de Gonzalo-Calvo et al. (2016)
STZ treated mice/HG treated primary cardiomyocyte	lncRNA-MIAT	lncRNA-MIAT lentivirus or CASP1 inhibitor/siMIAT (*in vitro* and *in vivo*)	miR-214-3p and IL-17	Promotes secretion of IL-17 and induces cardiac fibrosis	Qi et al. (2020)
HG treated cardiomyocyte of newly born mouse	lncRNA-Gas5	shRNA –Gas5 (*in vitro*)	miR−320−3p and Tcf-3	Promotes apoptosis	Su et al. (2020)
HG treated H9c2 cells and STZ treated mice	lncRNA-HOTAIR	Tail vein injection of AAV 2-HOTAIR and Ad-sh-HOTAIR (*in vitro* and *in vivo*)	miR-34 and Sirt1	Prevents cardiac inflammation, oxidative injury and apoptosis	Gao et al. (2019)
T2DM patients	hsa-circRNA11783-2	NA	NA	Coronary artery diseases	Li et al. (2017)
db/db mice	circRNAs_00203	rAd-circRNA_000203 (*in vitro*)	Col1a2 and CTGF, miR-26b-5p	Enhances expression of fibrosis associated genes	Tang et al. (2017)
HG treated cardiomyocytes/serum of diabetic patients	hsa_circ_0076631	Anti-sense oligonucleotide for hsa_circ_0076631 (*in vitro*)	miR-214-3p, caspase-1	Induces pyroptosis	Yang et al. (2019)

*ncRNA, non-coding RNA; miR, micro RNA; GLUT4, glucose transporter type 4; KLF15, Kruppel-like factor; HG, high glucose; STZ, streptozotocin; SGK1. serum- and glucocorticoid-regulated kinase 1; IGF1R, insulin-like growth factor-1 receptor; MEF2, myocyte enhancer factor-2; Tg mice, transgenic mice; FN1, fibronectin; COL4A1, collagen 1 alpha 1V; TGF-β, transforming growth factor-β1; SD rats, Sprague–Dawley rats; DUSP1, dual-specific phosphatase-1; JNK1, jun-amino-terminal kinase 1; ERK, extracellular signal-related kinase; Rac1, Ras-related C3 botulinum toxin substrate 1; AMPK, AMP-activated protein kinase; PGC-1α, peroxisome proliferator-activated receptor gamma coactivator 1-alpha; NLRP3, NLR family pyrin domain containing 3; HFD, high fat diet; Sirt1, sirtuin1; MMEC, myocardial microvascular endothelial cells; T2DM, type 2 diabetes mellitus; IGF2, insulin-like growth factor 2; LKB1, liver kinase B1; CD36, cluster of differentiation 36; VEGF, vascular endothelial growth factor; SPRED-1, sprouty related EVH1 domain Containing 1; lncRNA, long non-coding RNA; MALAT1, metastasis associated lung adenocarcinoma transcript 1; Crnde, colorectal neoplasia differentially expressed; MIAT, myocardial infarction associated transcript; LIPCAR, long intergenic non-coding RNA predicting cardiac remodeling; shRNA, short hairpin RNA; VDAC1, voltage-dependent anion-selective channel 1; AAV, adeno associated virus; Smad3, mothers against decapentaplegic homolog 3; IL17, interleukin 17; Gas 5, growth arrest specific 5; Tcf 3, transcription factor 3; HOTAIR, HOX antisense intergenic RNA; Circ-RNA, circular RNA; Col1a2, collagen alpha-2(I); CTGF, connective tissue growth factor; NA, not available.*

### Micro-RNAs in Diabetic Cardiomyopathy

Micro-RNA consists of highly conserved, single-stranded ncRNAs and range 16–27 nucleotides in length (Wahid et al., 2010; Fang et al., 2013; Joladarashi et al., 2015; Verma et al., 2017a,b; Lu and Rothenberg, 2018). They were first reported in nematode *Caenorhabditis elegans* (Lee et al., 1993). Biogenesis of miRNA is regulated by two nuclear ribonuclease III (RNase III) proteins, i.e., Drosha and Dicer. At the transcriptional level, miRNA’s biogenesis is regulated by Drosha, which cleaves the pre-miRNA and releases approximately 70–100 nucleotides of precursor miRNAs. After its transportation to the cytoplasm, Dicer cleaves the precursor and forms a mature miRNA. A significant function of miRNAs is to modulate transcription and translation programs (Ha and Kim, 2014).

Recent studies highlight a key role for miRNAs in cardiac disease (Van Rooij et al., 2008; Guo and Nair, 2017). Another study on cardiac myocytes demonstrated that the overexpression of miRNA-133 inhibits GLUT4 expression and suppresses the insulin-stimulated glucose uptake (Horie et al., 2009). Another study identified miR-200b regulating endothelial to mesenchymal transition (End MT) in mouse models of DMCM (Feng et al., 2016). Liu et al. (2014) demonstrated that cardiac fibroblasts subjected to hyperglycemic conditions (30 mM) exhibited an increase in miR-21 levels, further showing that increased miR-21 levels promoted collagen synthesis by targeting dual-specificity phosphatase 8 (DUSP8). Another study by Feng et al. (2010) performed on STZ-induced T1DM mouse models demonstrated significantly reduced levels of miR-133a in cardiac tissue. MiR-133a is an enriched muscle and has a role in muscle development and cellular differentiation (Liang et al., 2007). Chen et al. (2014) reported the involvement of miR-133a in diabetes-induced cardiac fibrosis, finding significant repression of miR-133a in the STZ-induced diabetic hearts, whereas the major markers of fibrosis [fibronectin 1 (FN1), tumor growth factor-β (TGF-β), and connective tissue growth factor] were found to be higher. Another study performed by Nandi et al. (2016) revealed the involvement of miR-133a in diabetic heart disease. They have shown miR-133a to mimic treatment in STZ rats, improving contractility of heart with subsequent upregulation of tyrosine hydroxylase, norepinephrine, and β-adrenergic receptor (Nandi et al., 2016). MiR-200c played an important role in cardiac hypertrophy and was found significantly higher in diabetic hearts. In diabetes-associated cardiac hypertrophy, a higher expression of miR-200c is reported, which negatively regulates the expression of dual-specificity phosphatase-1 (DUSP-1) (Singh et al., 2017). However, the miR-200c inhibition improves the level of DUSP-1 via suppressing the expression of the phosphorylated extracellular signal-regulated kinase (ERK), c-Jun N-terminal kinases (JNKs), and p38, further improving cardiac hypertrophy in high glucose (HG)-treated (30 mmol/L) rat cardiomyocytes (Singh et al., 2017). Recent findings by Tao et al. (2020) reveal the role of miR-144 in the protection of the diabetic heart by regulating cardiomyocyte apoptosis, displaying that miR-144 was found to be significantly decreased in HG-treated cardiomyocytes and in the heart of STZ-induced diabetic mice, which were associated with mitochondrial dysfunction and cell death. However, the overexpression of miR-144 ameliorates the mitochondrial dysfunction and negatively regulates cell death (Tao et al., 2020). Xu et al. (2020) established the role of miR-223 in DMCM. Cardiomyocytes subjected to HG showed elevated miR-223 levels. Further inhibition of miR-223 rescued the myocardial apoptosis and fibrosis by suppressing the expression NLRP3 inflammasome (Xu et al., 2020). Interestingly, Tang et al. (2018) showed the involvement of miR-22 in amelioration of DMCM. The overexpression of miR-22 reduced oxidative stress in DMCM by targeting sirtuin-1 (Tang et al., 2018). Using a high-fat-diet (HFD) mouse model, Copier et al. (2017) showed at 16 months that circulating miRNA (miR-19b-3p and miR-181b-5p) levels correlated with the development of cardiomyopathy. Furthermore, this study established miR-19b and miR-181b as potential biomarkers for DMCM. A recent study performed on myocardial microvascular endothelial cells (MMECs) from T2DM Goto-Kakizaki rats showed that miRNA-193-5p plays an important role as an angiogenic factor in DMCM by negatively regulating the expression of insulin growth factor-2 (IGF-2) (Yi et al., 2017). In another very intriguing study performed by Kuwabara et al. (2015), in HFD-fed C57BL/6 T2DM rat model, the expression of miRNA-451 levels was high in the myocardium. Further, cardiomyocyte-specific knockout of miRNA-451 led to an improvement in cardiac hypertrophy response compared with control mice. This finding established the potential role of miRNA-451 in DMCM by suppressing the liver kinase B1 (LKB1)/AMP-activated protein kinase pathway (Kuwabara et al., 2015). A recent study highlighted the importance of miR-320 in DMCM (Li et al., 2019). In this study, the authors reported upregulation of miR-320 in the db/db hearts and failing human hearts with pre-existing diabetes. To understand the functional role of miR-320, the authors performed gain and loss of miR-320 in db/db mouse and observed that miR-320 silencing improved cardiac dysfunction (Li et al., 2019), whereas miR-320 overexpression worsened the cardiac phenotype. Mechanistically, miR-320 regulates transcription of fatty acid metabolic genes to rescue lipotoxicity in the diabetic heart (Li et al., 2019). In a similar line, an intriguing study by Lew et al. (2020) demonstrated that early exercise in diabetes mice (from 8 weeks) strikingly rescued the onset and progression of DMCM. Importantly noted, diabetic mice did not exhibit any functional benefits when the exercise was initiated after the establishment of cardiac dysfunction (at 16 weeks). Mechanistically, diabetic mouse hearts (16 weeks) with cardiac dysfunction exhibited repression of miRs, viz., miR-126 (proangiogenic), miR-499 (anti-angiogenic), miR-15a/b, and miR-133 (anti-fibrotic). The findings by Lew et al. (2020) suggest that a downregulation of these miRs leads to pathological remodeling in the diabetic heart. Interestingly, early exercise normalizes the aforementioned miR expression. Furthermore, silencing of miR-126 abrogated exercise-mediated rescue of DMCM. Overall, these results highlight the importance of physical exercise in protecting CV health in patients with diabetes (Lew et al., 2020).

### Long Non-coding RNAs in Diabetic Cardiomyopathy

Long non-coding RNAs are a class of ncRNAs with wide range of lengths ranging from 200 to over 100 kb (Ulitsky and Bartel, 2013; Li J. et al., 2016; Kopp and Mendell, 2018; Palazzo and Koonin, 2020; Tariq et al., 2020). LncRNAs can be classified into (a) stand-alone RNAs, (b) antisense transcripts, (c) long intron ncRNAs, (d) pseudogenes containing lncRNA, and (e) other lncRNAs with divergent transcripts. LncRNAs can be found in the nucleus and cytosol. They participate in many biological functions like transcriptional regulation, cellular trafficking, nuclear compartmentalization, apoptosis, genomic imprinting, and cell cycle (Goyal et al., 2018). Previously published reports demonstrate that lncRNAs exhibit a significant role in various pathophysiological conditions of CV disorders, including DMCM via targeting miRNA/mRNA or proteins (Zhang et al., 2019).

A recent study performed on diabetic rats showed elevated lncRNAs, including MALAT1, in an STZ model of DMCM. In this study, a knockdown of lncRNA MALAT1 led to an improvement in LV function by reducing cardiomyocyte apoptosis (Zhang et al., 2016). Li X. et al. (2016) recently demonstrated the role of lncRNA H19 in DMCM. They found that H19 levels were downregulated in diabetic patients’ myocardium (Li X. et al., 2016). The rescue of H19 expression reduced inflammation, oxidative stress, and apoptosis, leading to an improvement in LV function in STZ-induced SD rats. To tease out a molecular mechanism, Li X. et al. (2016) showed an overexpression of H19, which produces a reduction in VDAC1 expression and apoptosis in HG-treated (30 mmol/L) cardiomyocytes. This study established a possible novel lncRNA therapeutic approach for treatment of DMCM. An interesting finding by Zheng et al. (2019) reveals that the role of cardiac-specific lncRNA-Crnde (colorectal neoplasia differentially expressed) is negatively associated with cardiac fibrosis in an HFD and low-dose STZ-treated mice. Using the *in vitro* model system demonstrated that Crnde expression inversely correlates with myofibroblast differentiation, leading to cardiac fibrosis. In corroboration with *in vitro* findings, *in vivo* adeno-associated virus-mediated overexpression of Crnde significantly reduced fibrosis. This finding establishes lncRNA therapeutics’ potential role against cardiac fibrosis concerning DMCM (Zheng et al., 2019).

Recent findings by Yang et al. (2018) explore the role of ncRNA-Kcnq1ot1 in cardiac pyroptosis in DMCM. This study found elevated levels of Kcnq1ot1 in cardiomyocytes treated with HG (50 mmol/L) and in cardiac tissue obtained from STZ-induced diabetic mice. Significantly, lncRNA-Kcnq1ot1 knockdown lowered cardiomyocyte and cardiac cell death and improved cardiac function *in vivo*. These findings suggest that Kcnq1ot1 could be a potential therapeutic target in DMCM (Yang et al., 2018). Another recent discovery by Qi et al. (2020) revealed that HG-treated cardiomyocytes increased lncRNA-MIAT expression leading to induction of pro-inflammatory cytokine interleukin-17 (IL-17). IL-17 production further promotes inflammation in the heart and subsequently leads to cardiac fibrosis (Qi et al., 2020). A recent report showed lncRNA-Gas5 involvement in apoptosis post hyperglycemia in DMCM neonatal cardiomyocytes (Su et al., 2020). Su et al. (2020) reported that the expression of lncRNA-Gas5 increased significantly in a time-dependent manner in HG-treated newly born cardiomyocytes, which subsequently leads to apoptosis. Su et al. (2020) found the rescue of apoptosis when they inhibited the lncRNA-Gas5. Gao et al. (2019) reported the role of the HOX transcript antisense RNA (HOTAIR) lncRNA in DMCM, demonstrating that the overexpression of HOTAIR in cardiomyocytes of STZ-treated mice attenuated the cardiomyocyte death and improved oxidative stress, inflammation, and cardiac function, further investigating the role that HOTAIR plays in *in vitro* hyperglycemic treated H9c2 cells and finding increased apoptosis, inflammation, and oxidative injury.

Other studies have shown an essential role of lncRNAs. LIPCAR is positively associated with grade I diastolic dysfunction (de Gonzalo-Calvo et al., 2016). SENCAR and MIAT were related to cardiac remodeling observed via LV mass to LV end-diastolic volume ratio. This study highlights the role of lncRNAs as potential predictors of diastolic function in diabetic patients (de Gonzalo-Calvo et al., 2016).

### Circular RNAs as Novel Therapeutic Targets for Diabetic Cardiomyopathy

Circular RNAs were discovered by Sanger et al. (1976) and are “covalently closed single-stranded circular RNA molecules.” These classes of RNAs are highly abundant in eukaryotes, and many of them are evolutionary conserved. In respect of other forms of spliced linear RNA, circRNAs are formed by the joining of downstream 5′ splice site to an upstream 3′ splice site, making them covalently circularized RNA loops (Gong et al., 2019). They are derived from exons or introns or a combination of exon/intron or lariats of size ranging 100 nucleotides to 4 kb (Lasda and Parker, 2014). Due to their unique structure, circRNAs are naturally insusceptible to ribonuclease R (RNase R), which results in their increased half-life (Kishore et al., 2020). These abilities make circRNAs potent biomarkers for various diseases, including heart diseases and diabetes. We have recently demonstrated that circFndc3b expression rescue promoted cardiac repair and improved cardiac function regulating the FUS/VEGF-A axis (Garikipati et al., 2019). A recent report demonstrated a correlation between circRNA (Hsa-circRNA11783-2) expression, coronary artery disease, and T2DM (Li et al., 2017). Using a db/db mouse subjected to Ang-II stress, Tang et al. (2017) identified upregulation of circRNA_00203, further displaying that overexpression of circRNAs_00203 enhanced fibrosis-related genes Col1a2 and CTGF via miR-26b-5p sponging in isolated cardiac fibroblasts. These studies point out the importance of circRNAs in diabetes-induced cardiac fibrosis (Tang et al., 2017). Yang et al. (2019) recently showed increased hsa_circ_0076631 levels in diabetic patients and cardiomyocytes under hyperglycemic stress. *In vitro* knockdown of hsa_circ_0076631 in cardiomyocytes abolished HG-induced apoptosis via miR-214-3p sponging and inhibiting caspase-1 expression. These results highlight the role of hsa_circ_0076631 regulating pyroptosis in diabetic cardiomyocytes. Given the increasing knowledge on the association of circRNAs in DMCM, there is enormous potential for further exploration of additional mechanisms and novel therapeutic targets.

## Conclusion and Future Directions

We summarize that ncRNAs play an important role in DMCM. Of the ncRNAs focused in this review, miRNAs are the most studied in the context of CVD. Tremendous progress on miRNA-based therapeutics led them to clinical trials. In a phase 1 clinical trial, CDR1321 (a novel synthetic miR-92a inhibitor) was used to treat HF^1^ (unique identifier: NCT04045405). A phase 1 clinical trial has recently been initiated using RG-102 (miR-21 inhibitor) to treat kidney fibrosis (see text footnote 1; unique identifier: NCT03373786). This is encouraging; however, significant hurdles with miRNA therapeutics include delivering miRNA inhibitors to specific organs/cells and associated off-target effects.

Circulating ncRNAs including miRNA (miR-320), lncRNA (lncRNA-LIPCAR, SENCAR, and MIAT), and circRNA (Hsa-circRNA11783-2 and hsa_circ_0076631) were shown to have diagnostic and prognostic values (de Gonzalo-Calvo et al., 2016; Li et al., 2017, 2019; Yang et al., 2019). Given that the studies performed were in a small patient cohort, future studies should focus on a larger number of patients, multi-centric trials, racial backgrounds, and different time points after diabetes.

While the field of long ncRNAs (lncRNA and circRNAs) is emerging in the context of DMCM, for a comprehensive understanding of lncRNAs in DMCM, the following issues need to be addressed: (a) robust computational techniques for their identification and downstream targets should be standardized, (b) novel regulatory mechanisms need to be uncovered (apart from their miRNA/RNA binding protein interaction), (c) it should be investigated if lncRNA translation products proteins/peptides are functional, and (d) genetic models of lncRNA should be established.

Thus, identification, characterization, and mechanisms of ncRNAs (especially lncRNA and circRNAs), which play a regulatory role in the network of interactions governing DMCM, are fundamental pieces of the puzzle that need to be solved to allow better treatment and prevention of DMCM.

## Author Contributions

AR, RG, and VNSG conceived the study. AR, RG, and DR collected the material and wrote the manuscript. MK and VNSG provided critical feedback and helped to shape the manuscript. BL contributed towards manuscript revision and rewriting few sections during the revision of manuscript. All authors contributed to the article and approved the submitted version.

## Conflict of Interest

The authors declare that the research was conducted in the absence of any commercial or financial relationships that could be construed as a potential conflict of interest.

## Footnotes

1 https://www.clinicaltrials.gov
